# Next-Generation Dental Materials: Exploring Bacterial Biofilm Formation on 3D-Printable Resin-Based Composites

**DOI:** 10.3390/jfb16010012

**Published:** 2025-01-03

**Authors:** Emerson Koji Uehara, Gustavo Castro de Lima, Janaina de Cassia Orlandi Sardi, Luciene Cristina de Figueiredo, Jamil Awad Shibli, Thabet Asbi, Doron Haim, José Augusto Rodrigues

**Affiliations:** 1 Dental Research Division, Guarulhos University, Guarulhos 07023-070, SP, Braziljashibli@yahoo.com (J.A.S.); 2 Maccabi-Dent Research Department, Tel-Aviv 6801298, Israel; 3 Department of Periodontology, Rambam Health Care Campus, Haifa 3109601, Israel

**Keywords:** periodontal pathogens, antimicrobial activity, periodontal disease, 3D printed resins, additive manufacturing, computer-aided design

## Abstract

This study evaluated the microbial growth profile of subgingival multispecies biofilm on 3D-printable resin-based composites (PRBCs). A 96-well cell plate cultivated a 39-species biofilm associated with periodontitis over 7 days. Cylindrical specimens with 12 mm high and 3 mm diameters were prepared by the PRBC group (Cosmos Temp-Yller; Prizma 3D Bio Crown; Prizma 3D Bio Prov) and an acrylic resin as control. Further, these specimens were immersed in the well plate to allow biofilm formation. After growing for 7 days, the metabolic biofilm activity was evaluated by colorimetric assay and the microbial profile by DNA-DNA hybridization. Kruskal–Wallis and Mann–Whitney tests evaluated each bacteria count and complex group. A greater biofilm formation was observed on PRBC groups than on acrylic resin. The microbiological profile of PRBC was associated with a less pathogenic biofilm, with an absence of a red complex. Acrylic resin showed low biofilm growth, but the biofilm profile was related to periodontal disease, characterized by red-complex bacteria. The selection of PRBC may contribute more effectively to maintaining periodontal health than acrylic resin.

## 1. Introduction

Conventional acrylic resins remain the most widely used polymeric materials in restorative dentistry. Computer-aided design (CAD) and computer-aided manufacturing (CAM) technologies have revolutionized the clinical workflow in dental practices. These technologies are divided into two main approaches: subtractive methods, such as milling, and additive methods, often referred to as 3D printing, which facilitate the production of 3D-printed restorations [1].

These CAD/CAM technologies present viable alternatives to traditional acrylic resin restorations. Subtractive milling methods, which shape prostheses from prefabricated blocks, are known for their high mechanical performance, accuracy, and favorable biological properties [2]. However, they face challenges in manufacturing complex geometries and involve significant material and tool consumption [3,4]. In contrast, additive methods like 3D printing avoid issues related to tool wear, offer efficient material use, and enable the fabrication of complex structures layer by layer, such as hollow internal designs. As a result, the popularity of 3D printing is increasing due to its advantages, including shorter production times, improved material efficiency, enhanced aesthetic, durability, and biocompatibility properties, enabling the production of temporary and permanent crowns, bridges, and inlay–onlay restorations [5,6].

The formation of pathogenic biofilms on dental surfaces is a critical factor contributing to secondary caries and periodontal diseases, which are primary causes of failure in indirect restorations [7]. The accumulation and maturation of bacterial biofilms can compromise the longevity of these restorations, as they serve as a reservoir for microorganisms that produce acids and proteolytic enzymes. These substances contribute to the degradation of both periodontal tissues and tooth structures, promoting the development of dental caries and periodontal disease [1,8].

Biofilm formation in the oral cavity begins with microorganisms like *Streptococcus mutans* and *Streptococcus sanguinis*, recognized as early colonizers. These microbes attach to the acquired pellicle on the tooth surface and aggregate into clusters surrounded by an extracellular polysaccharide matrix, forming a biofilm that adheres firmly to the surface [9]. Supragingival biofilm primarily comprises Gram-positive bacteria, such as *Streptococci* and *Lactobacillus* sp. In contrast, subgingival biofilms are predominantly Gram-negative anaerobic bacteria, including *Fusobacterium nucleatum* and *Porphyromonas gingivalis*, known for their pathogenic potential in periodontal disease [8,9,10].

The interaction between biofilms and restorative materials, particularly 3D-printable resin-based composites (PRBCs), has significant interest due to variations in surface chemistry and roughness of these materials. Different resin and filler compositions, along with their size and shape, influence the adhesion and growth of biofilms [11]. Recent in vitro studies utilizing standardized models have simulated oral conditions, allowing for the development of both monospecies and multispecies biofilms that mimic in vivo conditions regarding growth rates, composition, and structure [12,13,14]. These models are invaluable for assessing the interaction between biomaterials and biofilms, providing insights into how variations in material properties affect biofilm formation and potentially the risk of secondary caries or periodontal disease.

Studies emphasize the clinical significance of controlling subgingival biofilms as part of periodontal therapy, illustrating that effective biofilm management can reduce the pathogenic load and improve periodontal outcomes [8]. Therefore, understanding the dynamics of biofilm formation on dental materials, especially newer technologies like 3D-printable RBCs, is crucial for developing strategies to mitigate their impact on dental restorations and treatment success [15].

Bacterial colonization plays a key role in biofilm formation and heightens the risk of periodontal infections. However, there are limited scientific data on the susceptibility of 3D-printed resin restorations to bacterial adhesion and biofilm development. Therefore, this study compares bacterial growth in a multispecies biofilm model across three types of 3D-printed resin-based composites. The null- hypothesis (h_0_) was that all materials were harbored equally by the bacterial specimens.

## 2. Materials and Methods

### 2.1. Experimental Design

The development of biofilm formation was studied in four indirect PRBCs: priZma 3D Bio Crown, priZma 3D Bio Prov, Cosmos, and Clássica (Table 1). All materials were prepared according to the manufacturer’s instructions.

### 2.2. Printed Specimens

A DLP 3D printer (Flashforge 3D Printer; Zhejiang Flashforge 3D Technology Co., Ltd., Jinhua, China) was used to prepare cylindrical samples from G1, G2, and G3 PRBCs. The samples were produced following the manufacturer’s recommendations, with a 50 μm layer printing thickness and sizes of 12 mm high and 3 mm in diameter. Then, the samples were cleaned with 99% isopropyl alcohol. The post-polymerization process of the samples was carried out in an ultraviolet light device for 30 min.

To prepare the acrylic resin, a plastic cylindrical model 12 mm high and 3 mm in diameter was prepared, inserted in a flask with investment, and heated to melt away, leaving a mold space. A polymethyl methacrylate (PMMA) resin was mixed per the manufacturer’s instructions. The resin was packed into the mold space, and the packed flask was then subjected to a controlled heat-curing process (1 h). After curing, the flask was carefully opened to remove the newly formed specimen. The specimens were trimmed to remove any excess resin and adjusted.

### 2.3. Biofilm Formation

To develop the biofilm model [16], pure cultures of each bacterial species were prepared. Most species, including *Actinomyces* sp., *Streptococcus* sp., and *Fusobacterium* sp., were cultivated on tryptone soy agar supplemented with 5% sheep blood under anaerobic conditions (85% nitrogen, 10% carbon dioxide, and 5% hydrogen). Eubacterium nodatum was grown on fastidious anaerobic agar with 5% sheep blood, while Porphyromonas gingivalis was cultured on tryptone soy agar enriched with yeast extract, 1% hemin, 5% menadione, and 5% sheep blood. Tannerella forsythia was grown on tryptone soy agar with yeast extract supplemented with 1% hemin, 5% menadione, 5% sheep blood, and 1% N-acetylmuramic acid. The bacterial species used to produce the in vitro polymicrobial subgingival biofilm were previously described [14].

After 48 h of growth on agar plates, all species were transferred to conical tubes containing brain heart infusion (BHI) broth (Becton Dickinson, Sparks, MD, USA) supplemented with 1% hemin. Following 24 h of growth in BHI broth, the optical density (OD) at 600 nm was adjusted to 0.1, corresponding to approximately 10^8^ cells/mL for each species. The individual bacterial suspensions were then diluted and combined to create a final biofilm inoculum containing 10^4^ cells/mL of each species.

The multispecies biofilm model was established using a 96-well plate (Nunc; Thermo Scientific, Roskilde, Denmark). Each well received 150 µL of the inoculum and a printed or control specimen. The plates were incubated at 37 °C under anaerobic conditions. After 72 h of incubation, the plate covers were transferred to new 96-well plates containing fresh BHI broth supplemented with 1% hemin and 5% sheep blood. Biofilm development continued for an additional four days. After seven days of biofilm formation, the printed and control specimens were collected for analysis. Two independent experiments were conducted: metabolic activity was assessed (n = 2), and checkerboard DNA-DNA hybridization was performed (n = 5).

### 2.4. Analysis of Biofilm Metabolic Activity

The metabolic activity of the biofilm was quantified using 2,3,5-triphenyl tetrazolium chloride (TTC) (catalog number 17779; Analytical Fluka) and spectrophotometric analysis. TTC is widely used to differentiate metabolically active bacterial cells from inactive ones through its enzymatic reduction to 1,3,5-triphenyl red formazan (TPHP), a reaction catalyzed by cellular dehydrogenases. This process results in a color change in the substrate, which is measured spectrophotometrically and indirectly indicates bacterial metabolic activity. To evaluate the biofilm’s metabolic activity, samples were washed twice with a washing solution and then transferred to microplate wells containing 190 µL of fresh BHI medium supplemented with 1% hemin and 10 µL TTC solution (0.1%). The plates were incubated under anaerobic conditions at 37 °C for 24 h. The conversion of TTC to TPHP was measured by absorbance at 485 nm using a fluorescence spectrophotometer, the BioTek Epoch Microplate Spectrophotometer (Agilent Technologies, Santa Clara, CA, USA). As an experimental control, wells containing 190 µL of fresh BHI medium supplemented with 1% hemin and 0.1% TTC solution were used but without biofilm samples [14].

### 2.5. Checkerboard DNA-DNA Hybridization

Biofilm suspensions in plastic conical tubes were heated in a water bath at boiling temperature for 10 min and then neutralized with 0.8 mL of 5 M ammonium acetate. Each suspension containing free DNA was loaded into one of the 30 channels of the minislot apparatus (Immunetics, Cambridge, MA, USA) and transferred onto a positively charged nylon membrane (15 × 15 cm) (Amersham Biosciences Amersham, Buckinghamshire, UK). The last two channels served as controls, containing a mixture of target microorganisms at concentrations of 10^5^ and 10^6^ cells [17].

Following DNA transfer, the membrane was removed, and DNA was fixed by heating at 120 °C for 20 min. Prehybridization was carried out at 42 °C for 1 h in a solution comprising 50% formamide, 1% casein, 5× SSC, 25 mM sodium phosphate (pH 6.5), and 0.5 mg/mL yeast RNA.

The membrane was placed onto the Miniblotter 45 (Immunetics) for hybridization, with the DNA sample lines oriented perpendicular to the channels. A DNA probe diluted to 20 ng/mL was added to each channel in 130 mL of hybridization solution containing 45% formamide, 5× SSC, 20 mM sodium phosphate, 0.2 mg/mL yeast RNA, 10% dextran sulfate, and 1% casein. Hybridization was performed for a minimum of 20 h at 42 °C.

### 2.6. Detection of Species

After the hybridization period, the membrane was removed from the Miniblotter 45 (Immunetics) and washed for 40 min at 65 °C in an astringent solution containing 1% SDS, one mM EDTA, and 20 mM Na_2_HPO_4_ to eliminate non-specifically bound probes. The membrane was then immersed for 1 h in a blocking solution composed of 1% maleic acid (C_4_H_4_O_4_, Vetec, Duque de Caxias, Brazil), 3 M NaCl, 0.2 M NaOH (Labsynth, Diadema, Brazil), 0.3% Tween 20 (Vetec, Duque de Caxias, Brazil), and 0.5% casein, adjusted to pH 8.0. Subsequently, it was incubated for 30 min in the same solution containing an anti-digoxigenin antibody conjugated to alkaline phosphatase (Roche) at a 1:10,000 dilution.

The membrane was washed twice for 20 min in a washing solution of 0.1 M maleic acid, 3 M NaCl, 0.2 M NaOH, and 0.3% Tween 20, pH 8.0. It was then rewashed for 5 min in a solution of 0.1 M Tris-HCl and 0.1 M NaCl, pH 9.5.

The membrane was incubated for 45 min at 37 °C in a detection solution containing the CDP-Star™ substrate (Amersham) for alkaline phosphatase to detect hybridization signals. The membrane was placed in a 30 × 40 cm radiographic cassette (Konex, São Paulo, SP, Brazil) beneath an 18 × 24 cm radiographic film (Agfa Gevaert, NV Mortsel, Belgium) for approximately 40 min. The film was developed manually using the conventional temperature-time method, following the manufacturer’s instructions. The developing solutions (Kodak Brasileira Com. E Ind. Ltd.a, São José dos Campos, SP, Brazil) were maintained at 20 °C.

Radiographic films were analyzed by a single trained examiner calibrated and blinded to the experimental conditions. Each film was reviewed twice on different days to confirm consistency in the results. The signal intensity produced by probes in the biofilm samples was compared to the signals from control lanes containing 10^5^ and 10^6^ bacteria. A scoring system was used as follows:

- 0: No signal detected
- 1: Signal weaker than the 10^5^ cell control
- 2: Signal equivalent to 10^5^ cells
- 3: Signal intensity between 10^5^ and 10^6^ cells
- 4: Signal equivalent to 10^6^ cells
- 5: Signal exceeding 10^6^ cells

These scores were used to quantify and compare the levels of different bacterial species across the evaluated samples.

### 2.7. Statistical Analysis

Metabolic activity among groups was described as a percentage of control acrylic resin (G4). Microbiological data were presented as counts (levels) of 39 bacterial species evaluated. The experimental design included 40 samples (10 samples from each evaluated group). All experiments were performed in triplicate, and the median values were assessed for each group and then analyzed using a non-parametric test (Kruskall Wallis test).

The statistical procedures were performed using Jamovi 2.4.1.0 [18]. Outlier data were removed, and significant differences between specimens and groups were evaluated using Kruskal–Wallis and Mann–Whitney tests to assess each bacteria count and ANOVA to evaluate the proportions among complex groups. The comparison to determine statistical significance was set at 5%.

## 3. Results

Higher metabolic activity was observed on biofilm exposed to Cosmos Temp (131.9%) than conventional acrylic resin. The other PRBCs showed a similar metabolic activity with a light reduction on Prizma 3D Bio Prov and a slight increase on Prizma 3D Bio Crown (Table 2).

The data analysis of checkerboard DNA-DNA (Table 3) showed statistically significant differences in bacteria counts among groups of *A. gerencseriae*, *S. gordonii*, *S. oralis*, *C. gingivalis*, *C. sputigena*, *P. nigrescens*, *and P. gingivalis. C. gracilis*, *F. nucleatum.* ssp. *vincentii*, *P. intermedia*, *P. micra*, *and S. mutans* growth were not detected on specimens.

The analysis of the proportion of complexes among control and tested groups showed statistically significant differences in the Yellow, Orange, and Red complexes and Actinomyces (Table 4). Also, the highest total bacteria count was for Prizma 3D Bio Crown, which statistically differed from Clássico. The same result was observed for the total bacterial species count (Table 4).

## 4. Discussion

Numerous studies have focused on the effectiveness of novel definitive and interim indirect dental restorations, considering their complex chemical compositions, fabrication techniques, and interaction with soft tissues.

The formation of bacterial biofilms on restoration surfaces is closely linked to the initiation and progression of dental diseases, such as periodontitis and peri-implantitis. The results showed that the PRBCs studied could reduce bacterial growth. The methodology employed requires bacterial adhesion and colonization on PRBC specimens’ surfaces. Compared with other studies using the same polymicrobial biofilm model, the bacteria accounts were very low, and some were undetected, suggesting an antibacterial effect against most of the tested periodontopathogenic bacteria found around teeth and dental implants.

While earlier studies have documented minimal monomer release from materials fabricating indirect restorations [20], these materials may still release unpolymerized monomers due to physical and chemical interactions within the oral environment [21]. PRBCs can have a toxic effect due to the release of monomers from their structures, which jeopardizes bacterial colonization. The assessment of metabolic activity (TTC) showed that Cosmos Temp PRBC exhibited more significant activity than acrylic resin. Prizma 3D Bio Crown PRBC’s total counts were higher than those of acrylic resin by checkerboard DNA-DNA hybridization.

**Figure 1:**
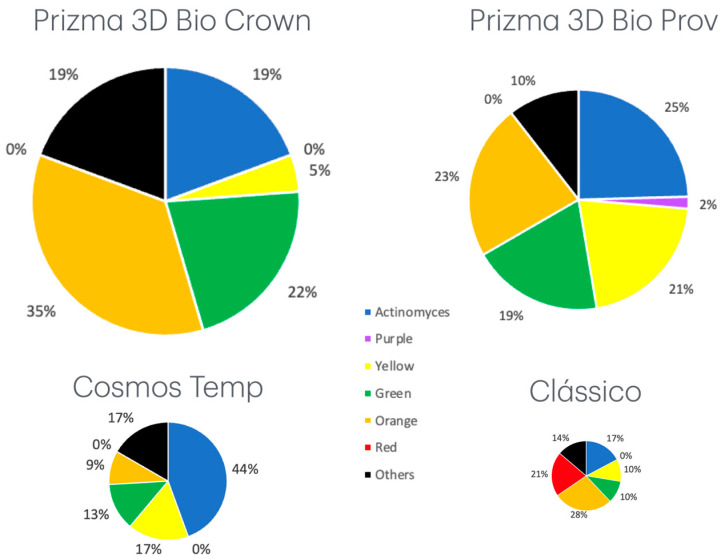
Pie charts describing the total counts of microbial complexes (×10^5^) and percentage detected by Checkerboard DNA-DNA hybridization. The size of the pie charts represents the proportion of total bacterial counts.

Although acrylic conventional resin showed a higher antibacterial effect, the specimens allowed the growth of red complex bacteria (Figure 1). This profile, with the presence of red complex bacteria, is associated with established periodontal disease. The profile of tested PRBCs showed more bacterial counts than acrylic resin but with a higher proportion of other complexes. It is close to the profile related to health, suggesting that PRBCs can be more suitable for maintaining periodontal health.

To fully understand the complexity of biofilm formation on polymer-based restorative composites (PRBCs), additional factors must be considered, including their topographical and chemical properties, which play a crucial role [22,23]. Studies investigating bacterial adhesion on polished PRBCs are limited. These studies indicate that acrylic resins from different manufacturers exhibit slight variations in surface roughness. For instance, Schubert et al. evaluated four PRBC materials and their influence on *Streptococcus mutans* and *Candida albicans* adhesion. Polished sample mean surface roughness values ranged from 0.064 to 0.091 μm. After two hours of incubation, a positive but statistically insignificant correlation was observed between surface roughness and microbial adhesion [24]. Similarly, Mazurek-Popczyk et al. (2022) demonstrated that *Streptococcus* sp., *Staphylococcus* sp., and *Candida* sp. are capable of forming biofilms on PRBCs used in 3D-printed temporary restorations [1].

Moreover, during the 3D printing process, unreacted monomers may remain on the resin surface due to interactions with the oxygen-inhibiting layer, even after standard photopolymerization. This necessitates a post-curing process to polymerize residual resin and enhance the material’s physical properties. In the present study, all samples were cleaned and treated with ultraviolet light to ensure maximum polymerization. However, this study did not assess the impact of polishing, which could significantly influence these materials’ mechanical and biological characteristics.

Additionally, in vivo, microbial adhesion patterns may differ from those observed in vitro. The salivary pellicle is a critical factor affecting bacterial adhesion in the oral environment. The saliva coating creates a more hydrophilic surface, which can promote the adherence of early colonizers such as streptococci [25]. It is also worth noting that while standardized cylindrical disks are commonly used in research for consistency, they may not fully replicate the effects of tooth-shaped restorations or their interactions with periodontal tissues. Although the evaluation of the biomaterial behavior after the adsorption of 39 species harbored in this polymicrobial biofilm is strengthened in this study, some limitations must be highlighted. This in vitro study presented some limitations, such as the absence of a post-production (final finishing process) and salivary pellicle that could modulate bacterial adsorption. The incubation time of the in vitro biofilm may also impact the final results.

Finally, we can extrapolate these results based on the surfaces and material characteristics of the evaluated materials. The mechanical and microbial behavior between the interaction of the in vitro polymicrobial periodontopatogenic biofilm and the restorative material is currently under active research [26,27]. Developing new restorative materials using 3D printed technologies and the latest raw materials is promising, mainly to produce complex geometries with mechanical strengths that allow using those materials and definitive or long-period [28]. Taken together, the evolution of the restorative material can overcome common mechanical and biological problems related to conventional milled materials. Further studies comparing PRBCs with zirconia, ceramic, and composite materials will increase the field’s knowledge, specifically correlating the harbored bacterial species with roughness and porosity properties. These features could guide the clinician in choosing the restorations made with PRBCs and their impact on the periodontal tissues, specifically in subjects with a history of periodontitis.

## 5. Conclusions

Although more significant biofilm formation was observed in the PRBC groups compared to acrylic resin, the microbiological profile of PRBC is associated with a less pathogenic biofilm characterized by the absence of red complex bacteria. Therefore, the selection of PRBC may help maintain periodontal health. Acrylic resin showed low biofilm growth, but the biofilm profile was associated with periodontal disease, marked by many red complex bacteria.

## Figures and Tables

**Table 1:** Materials studied, type, and components percentage by weight.

Material Manufacturer	Type	Components % by Weight
G1-Cosmos Temp Yller	Temporary 3D-PRBCs	Oligomers > 80, Photoinitiators < 5, pigment < 1
G2-Prizma 3D Bio Crown MAKERTECH LABS LTDA	Permanent 3D-PRBCs	Methacrylated Monomers >10%, Amorphous silica ≤ 8%, Urethane Dimethacrylate < 75%, Titanium Dioxide < 0.5%, Zirconia silanized < 2%, Ceramic Fillers (proprietary) < 15%, Diphenyl (2,4,6-trimethylbenzoyl)- phosphine oxide < 5%
G3-Prizma 3D Bio Prov–Provisórios MAKERTECH LABS LTDA	Temporary 3D-PRBCs	Acrylic Monomers 20–40%, Acrylic Oligomers > 50, Dye/Pigment ≤ 2, Photoinitiators ≤ 1
G4-Clássico	Acrilic resin	Liquid: methyl methacrylate monomer, tepanol; powder: copolymer methyl ethyl methacrylate, DBP, pigments, peroxides.

**Table 2:** Percentage of biofilm bacteria metabolic activity.

Material	N	Average	Standard Deviation	Percentage
G1-Cosmos Temp	2	0.259	0.103	131.9%
G2-Prizma 3D Bio Crown	2	0.215	0.057	104.6%
G3-Prizma 3D Bio Prov	2	0.196	0.048	95.6%
G4-Classico	2	0.205	0.040	100.0%

**Table 3:** Mean total counts of the bacterial species (×10^5^) cultured in multispecies biofilms categorized into complexes by Socransky et al., 1998 [19] evaluated in biofilms formed on control and test groups. Symbols * ^φ^ show statistical significance (*p* < 0.05). nd. = Non-detected bacterial signal.

Complex	Bacterial Species (ATCC)	Cosmos Temp	Prizma 3D Bio Crown	Prizma 3D Bio Prov	Clássico	*p*-Value
Actinomyces	*A. gerencseriae*-23860	0.55 *	0.55 *	0.55 *	0.01 ^φ^	0.008 *
	*A. israelli*-12102	nd.	nd.	0.05	nd.	-
	*A. naeslundii*-12104	nd.	nd.	nd.	0.03	-
	*A. naeslundii* II oris-43146	0.05	0.10	0.10	0.05	0.252
Purple	*A. odontolyticus*-17929	nd.	0.01	0.05	nd.	0.072
	*V. parvula*-10790	nd.	nd.	0.01	nd.	-
Yellow	*S. gordonii*-10558	0.10 *	0.01 ^φ^	0.05 *	nd.	0.005 *
	*S. intermedius*-27335	nd.	0.01	nd.	nd.	-
	*S. mitis*-49456	0.01	0.01	nd.	nd.	-
	*S. oralis*-35037	0.07^φ^	0.10 *	nd.	nd.	0.007 *
	*S. sanguinis*-10556	0.05	0.05	0.55	0.05	0.321
Green	*A. actinomycetemcomitans*-29523	0.05	0.05	0.01	0.05	0.644
	*C. gingivalis*-33624	0.10 *	0.05 *^φ^	0.50 ^φ^	nd.	0.040 *
	*C. ochracea*-33596	nd.	0.05	0.05	nd.	0.123
	*C. sputigena*-33612	0.02 ^φ^	0.52 *	nd.	nd.	0.046 *
	*E. corrodens*-23834	0.01	0.05	nd.	nd.	0.093
Orange	*C. gracilis*-33236	nd.	nd.	nd.	nd.	-
	*C. rectus*-*33238*	0.00	0.03	nd.	0.00	0.392
	*C. showae*-51146	0.05	0.05	0.10	nd.	0.072
	*E. nodatum*-33099	nd.	nd.	nd.	0.03	-
	*F. nucleatum*. ssp. *nucleatum*-25586	nd.	0.05	nd.	nd.	-
	*F. nucleatum*. ssp. *polymorphum* 10953	0.01	0.05	nd.	nd.	0.093
	*F. nucleatum*. ssp. *vincentii*-49256	nd.	nd.	nd.	nd.	-
	*F. periodonticum*-33693	nd.	nd.	0.02	nd.	-
	*P. intermedia*-25611	nd.	nd.	nd.	nd.	-
	*P. micra*-33270	nd.	nd.	nd.	nd.	-
	*P. nigrescens*-33563	0.08 *^φ^	1.00 *	0.50 *	0.02 ^φ^	0.018 *
	*S. constellatus*-27823	0.01	0.01	0.05	0.07	0.072
Red	*P. gingivalis*-33277	nd.	nd.	0.01 *	0.10 ^φ^	0.002 *
	*T. forsythia*-43037	nd.	0.01	nd.	nd.	-
Other	*E. saburreum*-33271	nd.	0.05	0.10	0.03	0.129
	*G. morbillorum*-27824	0.01	0.05	nd.	nd.	0.093
	*L. buccalis*-14201	0.10	0.50	0.05	nd.	0.058
	*N. mucosa*-19696	0.05	0.01	nd.	nd.	0.093
	*P. acnes*-11827	nd.	0.01	nd.	nd.	-
	*P. melaninogenica*-25845	0.03	0.01	nd.	nd.	0.392
	*S. anginosus*-33397	0.50	0.05	0.10	0.02	0.240
	*S. noxia* 43541-25175	nd.	nd.	0.05	nd.	-
	*Streptococcus mutans*-25175	0.01	0.01	nd.	0.02	0.026

**Table 4:** The groups’ mean and standard deviation of bacterial complexes (×10^5^). The colors refer to the different complexes described by Socransky et al., 1998 [19] and the blue group comprises some *Actinomyces* species. Symbols * ^φ^ show statistical significance (*p* < 0.05).

Complex	Cosmos Temp	Prizma 3D Bio Crown	Prizma 3D Bio Prov	Clássico	*p*-Value
Actinomyces	0.60 ± 0.57	0.65 ± 0.52	0.70 ± 0.70	0.08 ± 0.04	0.026 *
Purple	0.01 ± 0.01	0.01 ± 0.01	0.05 ± 0.07	0.01 ± 0.01	0.072
Yellow	0.23 * ± 0.10	0.15 *^φ^ ± 0.06	0.60 *^φ^ ± 0.56	0.05 ^φ^ ± 0.05	0.020 *
Green	0.18 ± 0.05	0.72 ± 0.72	0.55 ± 0.77	0.05 ± 0.05	0.099
Orange	0.13 *^φ^ ± 0.10	1.18 * ± 0.10	0.65 *^φ^ ± 0.77	0.14 ^φ^ ± 0.12	0.042 *
Red	0.01 ^φ^ ± 0.01	0.01 ^φ^ ± 0.01	0.01 ^φ^ ± 0.01	0.10 * ± 0.01	0.002 *
Other	0.23 ± 0.13	0.65 ± 0.75	0.30 ± 0.14	0.07 ± 0.10	0.257
Total	1.35 *^φ^ ± 0.07	3.35 * ± 2.25	2.85 *^φ^ ± 2.90	0.48 ^φ^ ± 0.23	0.033 *

## Data Availability

Data supporting the article are available upon reasonable request from the corresponding author.
